# NXP-2 positive dermatomyositis with marked dysphagia following an insect bite

**DOI:** 10.1016/j.clinsp.2024.100420

**Published:** 2024-07-10

**Authors:** Larissa M. Bombardi, Carla Alexandra Scorza, Josef Finsterer, Fulvio Alexandre Scorza

**Affiliations:** a Ministério do Desenvolvimento Agrário e Agricultura Familiar (MDA), Brazil; b Disciplina de Neurociência, Universidade Federal de São Paulo/Escola Paulista de Medicina (UNIFESP/EPM), São Paulo, SP, Brasil; c Neurology Neurophysiology Center, Vienna, Austria

Dermatomyositis is an autoimmune disease that belongs to the group of collagen diseases (Diffuse Connective Tissue Diseases [CTDs]).^1^ There are four main variants of dermatomyositis, classic, amyopathic, paraneoplastic, and juvenile dermatomyositis.^1^ Classic dermatomyositis is characterized by inflammation of the skin (“dermatous”) and the striated muscle (“myositis”).^1^ Among various skin manifestations, heliotrope rash and violaceous papules located at the interphalangeal or metacarpophalangeal joints (Guttron's papules) are the most common.^1^ Myositis presents with myalgia, proximal weakness, and elevated muscle enzymes.^2^

Dermatomyositis can be associated with either myositis-associated or myositis-specific autoantibodies, which are associated with different clinical phenotypes.^2^^,^^3^ The best known are ARS, anti-Mi-2, MDA5, TIF1-γ, and NXP-2/MORC-2 antibodies.^3^ NXP-2/MORC-3 is found in the nucleus and in nuclear dots known as Promyelocytic Leukemia (PML) nuclear bodies.^4^ A patient with NXP-2-associated dermatomyositis with severe dysphagia after an insect bite has not been reported.

The patient was an HIV-negative, senescent male who developed rash and myalgias in the lower legs a few days after a bite from an unknown insect, which spread to the thighs and later to the upper arms and shoulder muscles. The myalgias were so severe that they prevented movements of the legs and arms and performing simple daily activities. Myalgias were associated with dry mouth, erythema of the face, breast, and upper arms, and dysphagia. Clinical examination revealed a heliotrope rash on the face, upper arm and breast, with Gottron's papules on all fingers. Dermatomyositis was suspected based on the typical dermatological features and generalized myalgias. Blood tests revealed an increase in blood sedimentation rate, C-reactive protein, Creatine-Kinase (CK), transaminases, lactate dehydrogenase, and ferritin. Collagen-associated antibodies (antinuclear antibodies, SS-A/Ro antibodies, PCNA antibodies, and Ro-52 antibodies) were positive. Whole body FDG-PET-CT showed increased uptake in the upper arm and spinal muscle. Examination for neoplasia was negative. The myositis-specific antibody search was positive for NXP-2, Ro and Ro60 antibodies.

With prednisolone and methotrexate, myalgias and CK continued to decrease, but dysphagia continued to progress. MRI of the collum showed diffuse edema of the neck and pharyngeal muscles. Since the dysphagia did not improve with steroids and methotrexate, rituximab was started and a Percutaneous, Endoscopic Gastrostomy (PEG) was implanted.

The index patient is of interest for NXP-2-related classic dermatomyositis following an insect bite complicated by steroid- and methotrexate-refractory dysphagia. Dysphagia is a common presentation of NXP-2-related juvenile and adult dermatomyositis. In a study of 12 adult Indian patients with NXP-2-related polymositis (n = 7) and dermatomyositis (n = 5), dysphagia was reported in 5 (41.7 %).^5^ Only one patient with dermatomyositis had malignancy (breast cancer).^5^ In a study of 10 adult Italian adult patients with NXP-2-related dermatomyositis, 43 % developed dysphagia.^6^ In a study of 47 adult Japanese patients with NXP-2-related Idiopathic Immune Myopathy (IIM), polymyositis (n = 23), dermatomyositis (n = 24), 22 developed dysphagia.^7^ In a study of 56 adult patients with NXP-2-positive dermatomyositis, NXP-2-positive patients had a higher frequency of distal limb and neck weakness, dysphagia (62 %), myalgia, calcinosis, and subcutaneous edema compared to non-NXP-2 patients with dermatomyositis.^2^

Juvenile NXP-2-associated dermatomyositis with dysphagia has also been repeatedly reported.^5^^,^^7^^,^^8^ In a study of 9 juvenile patients with NXP-2-asosciated dermatomyositis, five of the included patients had dysphagia.^5^ None of these patients had malignancy.^5^ In a study of 29 juvenile patients with dermatomyositis, 37 % had dysphagia.^7^ Only one patient was diagnosed with malignancy.^7^ In a study of 26 Chinese patients with juvenile dermatomyositis, 15 suffered from dysphagia, hoarseness, or soft voice.^8^ In this study resistance to therapy was associated with edema, skin ulcers, muscle weakness (MRC < 4), CD4/CD8 ration > 1.4, and ferritin > 200 µg/mL.^8^ Six patients suffered from severe gastrointestinal involvement, five of whom died and one survived after Autologous Stem Cell Transplantation (ASCT).^8^ Risk factors for gastrointestinal involvement and mortality included edema, skin ulcers, dysphagia/dysarthria, Body Mass Index (BMI < 15), and ANA positivity.^8^ It was concluded that edema, skin ulcer, and severe muscle weakness predict treatment resistance, gastrointestinal involvement, and mortality in NXP-2-related juvenile dermatomyositis.^8^

In a systematic review and meta-analysis of 28 studies including 4764 IIM patients, NXP-2-related dermatomyositis was associated with an increased risk of developing five clinical features ‒ edema, muscle weakness, myalgia/myodynia, calcinosis, and dysphagia.^9^ The presence of NXP-2 antibodies was not associated with increased mortality in this meta-analysis.^9^ It was concluded that NXP-2-related dermatomyositis has different clinical presentations with unique features and prognoses.^9^ When comparing 60 patients with NXP-2-related dermatomyositis with 211 patients with NXP-2-negative myositis, dysphagia and myositis were more common in the NXP-2 positives cases.^10^ Dysphagia was also more common in a retrospective study of 27 patients with NXP-2-related dermatomyositis than in non-NXP-2-related myositis.^11^

Whether the insect bite caused dermatomyositis in the index patient remains speculative. Few reports of insect bites and dermatomyositis have been published. Fulminant fatal dermatomyositis and myasthenia gravis following a bee sting were reported in a Hungarian patient.^12^ One argument for the insect bite as the cause of dysphagia in the index patient is the absence of malignancy.

In summary, this case demonstrates that NXP-2-associated dermatomyositis can be associated with severe dysphagia that may not respond to steroids and requires more aggressive treatment.
